# Facing the escalating burden of dengue: Challenges and perspectives

**DOI:** 10.1371/journal.pgph.0002598

**Published:** 2023-12-15

**Authors:** Gathsaurie Neelika Malavige, Peter Sjö, Kavita Singh, Jean-Michel Piedagnel, Charles Mowbray, Sergio Estani, Steven Chee Loon Lim, Andre M. Siquierra, Graham S. Ogg, Laurent Fraisse, Isabela Ribeiro

**Affiliations:** 1 Drugs for Neglected Diseases Initiative, Geneva, Switzerland; 2 MRC Human Immunology Unit, MRC Weatherall Institute of Molecular Medicine, University of Oxford, Oxford, United Kingdom; 3 Ministry of Health, Putrajaya, Malaysia; 4 Fiocruz, Rio de Janeiro, Brazil; University of Ottawa Faculty of Medicine, CANADA

## Abstract

Dengue is the most rapidly emerging mosquito-borne infection and, due to climate change and unplanned urbanization, it is predicted that the global burden of dengue will rise further as the infection spreads to new geographical locations. Dengue-endemic countries are often unable to cope with such increases, with health care facilities becoming overwhelmed during each dengue season. Furthermore, although dengue has been predominantly a childhood illness in the past, it currently mostly affects adults in many countries, with higher incidence of severe disease and mortality rates in pregnant women and in those with comorbidities. As there is currently no specific treatment for dengue and no early biomarker to identify those who will progress to develop vascular leakage, all individuals with dengue are closely monitored in case they need fluid management. Furthermore, diagnosing patients with acute dengue is challenging due to the similarity of clinical symptoms during early illness and poor sensitivity and specificity of point-of-care diagnostic tests. Novel vector control methods, such as the release of *Wolbachia-*infected mosquitoes, have shown promising results by reducing vector density and dengue incidence in clinical trial settings. A new dengue vaccine, TAK-003, had an efficacy of 61.2% against virologically confirmed dengue, 84.1% efficacy against hospitalizations and a 70% efficacy against development of dengue haemorrhagic fever (DHF) at 54 months. While vaccines and mosquito control methods are welcome, they alone are unlikely to fully reduce the burden of dengue, and a treatment for dengue is therefore essential. Several novel antiviral drugs are currently being evaluated along with drugs that inhibit host mediators, such as mast cell products. Although viral proteins such as NS1 contribute to the vascular leak observed in severe dengue, the host immune response to the viral infection also plays a significant role in progression to severe disease. There is an urgent need to discover safe and effective treatments for dengue to prevent disease progression.

## Introduction

Dengue is the most rapidly growing mosquito-borne infection in the world and, due to the burden of disease, the World Health Organization named it as one of the top ten threats to global health in 2019 [1,2]. Global infections have been steadily increasing over the last three decades, with the global age-standardized death rate increasing from 0.31 per 100,000 population in 1990 to 0.53 per 100,000 population in 2017, with disability adjusted life years (DALYs) increasing by 109% [3]. However, the burden of dengue infections is predicted to rise further with an increase in vector densities and vector competence, rapid urbanization, population expansion, increased international travel, waste disposal, sanitation and evolution of the virus [4]. Furthermore, *Aedes* species are likely to circulate in many other regions due to ongoing climate change and it is predicted that an additional 4.7 billion individuals will be at risk of being infected with dengue by 2070 [2].

**Fig 1:**
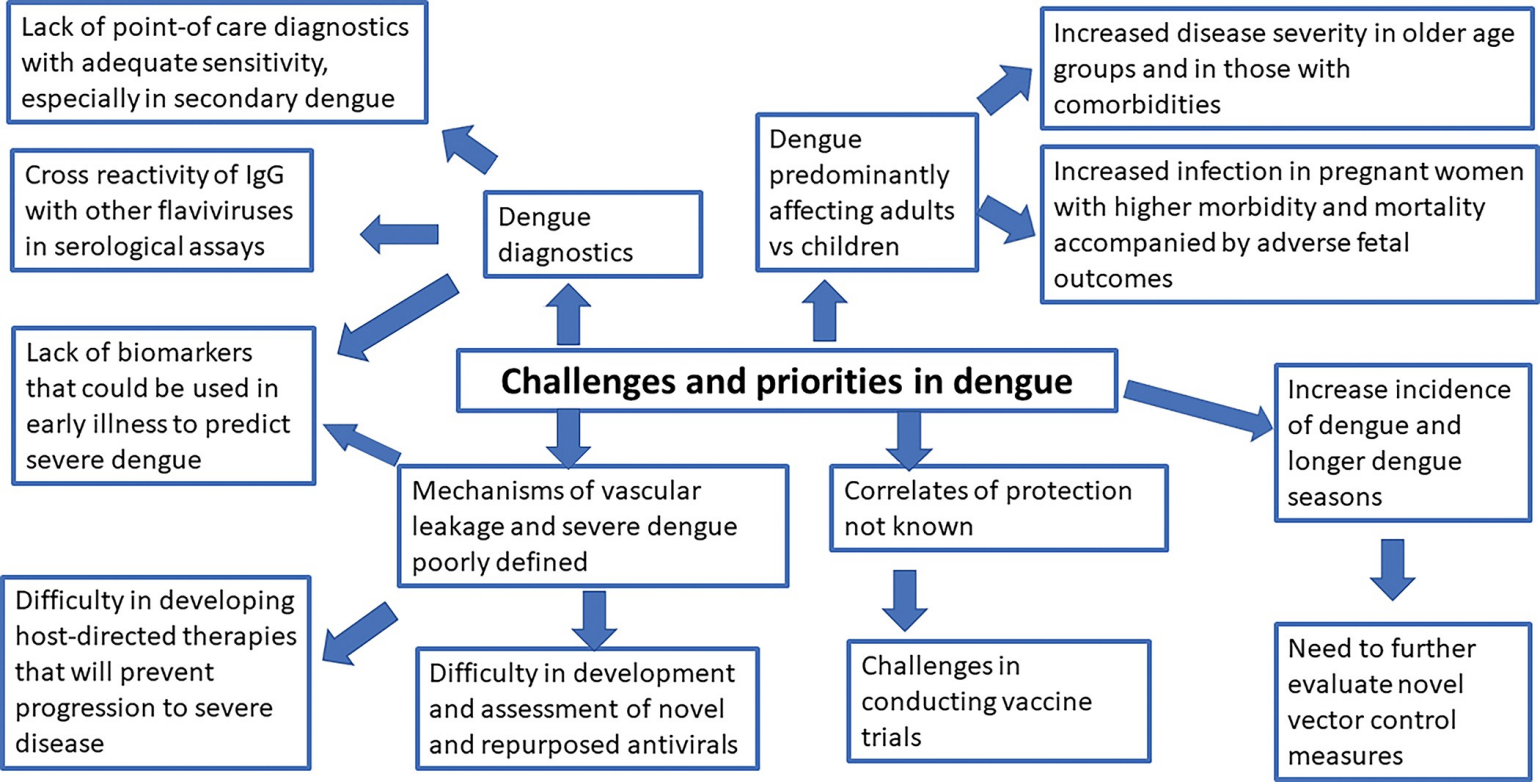
A summary of the challenges and priorities to reduce the increase in the burden of dengue.

There have been significant changes in the disease patterns in many endemic countries due to change in population demography, which brings new challenges. Although two vaccines have been licensed in some countries and there are emerging vector control strategies that may reduce the impact of dengue disease, there are no safe and effective specific drugs to treat patients with dengue and prevent disease progression. Furthermore, many of the point-of-care diagnostic tests available for NS1 antigen detection lack sensitivity, especially in patients with a secondary dengue infection, while the serological assays pose a challenge in interpretation due to the presence of cross-reactive IgG, in regions with co-circulation of multiple flaviviruses [5]. Due to the similarity of clinical features of many of the co-circulating arboviruses and with COVID-19 and influenza, many clinicians in dengue endemic countries face challenges to correctly identify patients who have dengue infection [6]. Although recent developments in dengue vaccines will hopefully reduce the burden of dengue, it is important to reflect on the lessons we have learnt during the COVID-19 pandemic. In this review, we discuss the likely impact of changes in vector densities, increases in infection rates, changes in population demographics and the use of vaccines on the landscape of dengue infections (Fig 1).

### Burden of infections in dengue-endemic countries

Dengue predominantly affects lower middle- and low-income countries, which often lack resources to handle the large number of dengue patients presenting to hospitals during dengue outbreaks. While many higher income countries were overwhelmed with COVID-19 admissions due to the more rapidly transmissible sub lineages of the omicron variant of SARS-CoV-2 [7], many dengue-endemic countries had to face the double burden of COVID-19 and dengue, although there were relatively fewer hospitalizations for COVID-19 due to vaccination [8,9]. With the ongoing threat of COVID-19, influenza and other arbovirus outbreaks [10], these countries will possibly face the challenge of treating patients with dengue, COVID-19, influenza and many other similar febrile illnesses simultaneously, which have similar clinical presentations during early illness [6].

The highest infection rates due to dengue are seen in South Asia, Southeast Asia and Latin America, with 70% of the burden of disease in Asia [11]. The incidence of dengue has continued to rise in these dengue endemic countries, with many countries in Asia and Latin America reporting a record number of cases in recent years [12]. Approximately 2.8 million cases of dengue cases reported in the Latin American region in 2022 with an incidence of 282.64 cases per 100,000 population. Co-circulation of all four DENV serotypes was reported, indicating high transmission rates [13]. Although dengue outbreaks initially were reported in Southeast Asia, gradually spreading to Latin America, current data show that the burden of dengue is similar in both regions [3].

Dengue has recently been documented to cause regular outbreaks in many areas in which it was not previously common, such as the Middle East, Africa, while smaller outbreaks were reported in Southern Europe [14,15]. Seroprevalence studies in many countries in Africa have demonstrated that dengue seroprevalence rates among healthy individuals varied widely from < 2% in regions in Kenya, Tanzania and Sudan, to > 25% in Burkina Faso, Cameroon, Ghana, Sao Tome and Sudan [15]. However, there are significant differences between the study designs and, therefore, significant differences in the seroprevalence rates reported in the same countries and different regions [15], highlighting the importance of carrying out well-designed seroprevalence studies in Africa. This information is essential to understand the true global burden of dengue and especially its burden in very neglected populations.

Dengue is a costly infection, with an estimated global cost of 8.9 billion USD in 2013 [16]. However, a subsequent study showed that the global estimations carried out by Shepard *et al*. had not considered indirect costs due to the number of working or school days lost, or the cost of outpatient visits. After considering the indirect costs mentioned above, it was estimated that the global costs due to loss of productivity, death and health care utilization was 39.3 billion USD in 2011 [17]. The actual costs of dengue are likely to be further underestimated due to under-reporting of cases. For instance, it was shown that India had approximately 53 million symptomatic infections in 2016 (282 times higher than the official reported numbers), with an estimated cost of 5.7 billion USD [18]. It was observed that the annual household days lost due to dengue in Malaysia ranged from 11.2 to 18.7 days, while symptomatic patients lost an average of 7.2 and 8.8 days of work for each infected person [19]. Therefore, it is evident that in addition to the morbidity and mortality due to dengue, the economic costs and societal impact of dengue are substantial. However, although housing conditions, economic status, and equity are likely to affect the disease burden and costs, these have not been considered in calculating the disease burden and costs, as there is limited data available [20].

### Changes in the clinical presentation and risk factors for severe disease

In the 1980s and 1990s, dengue was predominantly a childhood infection in many hyperendemic countries, with very few adults developing dengue haemorrhagic fever (DHF) and related complications. However, the mean age of reported symptomatic dengue infections and of those with DHF has increased steadily in many countries [21–23]. In Thailand, while the mean age of DHF was 8.1 years in 1981, it increased to 24.3 by 2017 [22]. In Sri Lanka, between 2000 and 2018, the proportion of cases dropped from 59.9% to 35.7% in children < 19 years of age and from 27.6% to 16% in children < 9 years of age [21]. While changes in population demographics in many Asian countries are likely to have contributed to this, it has been found that other factors, such as the susceptibility to multiple serotypes and changes in the extent of infection in the population could also have played a role [22,24]. It was demonstrated that due to intense dengue control programs in Singapore, the *Aedes* house index fell from 48% in 1966 to 1% in 1990, with a paradoxical increase in dengue cases and a rise in age of infection. The reduction in *Aedes* mosquitos led to reduction in the seroprevalence in the population and the extent of infection, which led to gradual shifting of the age of infection into adulthood [24]. Therefore, apart from changes in the population demography, the changes in extent of infection due to *Aedes* control measures in many countries could also have led to the shift of age of dengue infection. This change in the mean age of dengue infection poses very different challenges to dengue-endemic countries.

Diabetes, hypertension, renal disease, and cardiovascular disease are known risk factors for progression to severe disease and are also likely to increase the likelihood of hospitalization [25–27]. The prevalence of metabolic disease has been markedly rising in many Asian and Latin American countries [28,29]. Approximately 20% of the adult population in Asian countries have metabolic syndrome, with a steep rise in the prevalence of diabetes [29]. Due to this trend, since more dengue infections are seen in adults than in children, the proportion of individuals with dengue progressing to severe disease is likely to increase. Since concurrent bacterial infections in patients with severe dengue are also more frequent among patients with comorbidities [30,31], additional infections in patients with diabetes are likely to be more challenging to treat.

This increase in the number of dengue infections in adults also implies higher infection rates among pregnant women. Dengue infection in pregnancy is more likely to lead to severe disease and fatalities; for example, fatalities were 450 times higher in pregnant women with DHF compared to non-pregnant women [32]. Furthermore, pregnant women were more likely to require intensive care admission and ventilatory support and were more likely to have multiorgan failure [33]. DHF was the leading cause of maternal deaths in Sri Lanka in 2017, accounting for 16.5% deaths [34]. Maternal dengue is not only associated with poor maternal outcomes, but also with many adverse fetal outcomes, such as preterm delivery, fetal distress, stillbirth, and miscarriage [32]. In a large study in Brazil, maternal dengue, even when mild can lead to adverse fetal outcomes such as low birth weight and increased risk of hospitalization of children up to 3 years after birth [35]. Therefore, more data are needed to understand the effects of maternal dengue not only on the mother but also the fetus.

### Impact of novel vector control methods

The dengue viruses (DENVs) are transmitted by the *Aedes* species of mosquito, which also transmits many other viruses such as zika, chikungunya and yellow fever. The rise in global temperatures would lead to an increase the dengue burden in dengue endemic countries in the tropics and subtropics, while also resulting in outbreaks during warmer months in temperate climates, such as in Southern Europe and USA. Higher temperatures increase the biting frequency of mosquitoes and also increase the vector competence [36–38]. Increased temperatures result in a shorter extrinsic incubation period due to shorter gonadotropic cycles in mosquitoes, which leads to release of mature mosquitoes in a shorter period [37,39]. Furthermore, although *Aedes aegypti* is the primary vector that transmits dengue, increase in temperature also increases the competence of *Aedes albopictus* in transmitting dengue [40,41]. Improper waste disposal, collection of plastic and porcelain containers in waste and maintaining solid waste for longer than seven days, have contributed to was shown to increase *Aedes aegpti* breeding and therefore, increase in transmission [42,43]. Collectively, climate change, urbanization, improper waste disposal, and increase in travel are likely to lead to a significant increase in dengue infections globally.

However, there are many novel vector control methods under evaluation. Conventional methods relied on prevention of household-level contact between the vector and host by using mosquito nets, repellents and spraying with insecticides, but these have been difficult to implement, and the mosquitoes can develop resistance to many insecticides [44]. Novel biotechnology-based techniques, such as infecting the mosquitoes with *Wolbachia*, the release of sterile mosquitoes, and use of spatial repellents have shown promising results [45–47]. The introduction of the bacterium *Wolbachia* to *Aedes aegypti* impairs the ability of the DENV to replicate within the vector and thereby curtain DENV transmission [48]. *Wolbachia* has shown to act by stimulating innate immune pathways in *Aedes aegpti* thereby inhibiting virus replication [49]. The release of *Wolbachia*-infected mosquitoes significantly reduces symptomatic dengue and hospitalizations in Indonesia [46]. Therefore, the use of *Wolbachia*-infected *Aedes* mosquitoes shows potential for significantly reducing the burden of dengue and possibly halting dengue transmission in many disease endemic countries. However, sustainability of this method would depend on the possibility of development of resistance by the virus. As the DENV is an RNA virus, and as it undergoes a more frequent mutation rate than *Wolbachia* and *Aedes aegypti*, there is a potential of resistance strains emerging [50].

In addition to the *Wolbachia* infected mosquitoes, the use of spatial repellents is been investigated [47,51]. A trial in Peru showed that the use of spatial repellents significantly reduced the *Aedes aegypti* abundance by 28.6% and the incidence of vector transmitted infection by 34.1% [47]. Trials evaluating spatial repellents are currently being carried in other sites, such as in Sri Lanka [51], and if found effective, this would be a cheap, easy to use technique for vector control. While these methods have the potential to reduce the burden of dengue in disease-endemic countries, their effectiveness in the context of increasing vector densities due to climate change and in the case of adaptation to the effects of *Wolbachia* is not known and should be further studied.

### Challenges in understanding dengue pathogenesis and correlates of protection

Although most individuals who are infected with the DENV develop asymptomatic or mild illness, a proportion develop severe disease manifestations in the form of DHF, organ dysfunction and severe bleeding. Vascular leak is the hallmark of severe dengue, which occurs due to endothelial dysfunction. The clinically detectable vascular leakage that occurs during dengue lasts for 24 to 48 hours and is completely reversible [52]. Therefore, it is evident that inflammatory mediator/s are likely to be important contributors to endothelial dysfunction. Although there have been many studies that have been conducted to identify inflammatory markers during early illness that predict subsequent development severe dengue, such studies have been limited to testing a few inflammatory mediators identified in smaller studies [53,54]. Furthermore, many adequately powered studies have not investigated such markers in early illness and also during the critical phase where leakage occurs. The lack of understanding of the exact mediator/s that lead to vascular leakage has hampered development of therapeutic targets and also in developing a reliable biomarker that could predict those who are likely to progress to develop leakage during early illness. As the clinical course of dengue is very dynamic, it would be important to investigate inflammatory mediators that could cause leakage in the febrile phase and critical phase in an unbiased manner.

Those with a secondary dengue infection and with comorbidities (diabetes, obesity, kidney disease) are at a higher risk of developing severe dengue. Antibody dependent enhancement (ADE) has been suggested to be an important mechanism of severe dengue during a secondary dengue infection. Following a primary infection, individuals develop type-specific neutralizing antibodies that usually provide lifelong protection [55]. However, some individuals also develop varied amounts of cross-reactive non-neutralizing antibodies that lead to ADE in subsequent infections [55]. A large longitudinal study in Nicaragua showed that lower levels of DENV specific antibodies did not necessarily enhance infection. They showed that while higher levels of neutralizing antibodies did protect against infection and severe disease, intermediate levels, appeared to lead to more symptomatic and severe disease manifestations [56]. Furthermore, both primary and secondary infections were equally likely to result in inapparent infections [57]. Since most secondary dengue infections are also mild or asymptomatic, the characteristics of antibody responses that lead to ADE and the reasons for development of such antibodies in some individuals is not clear. This lack of knowledge of correlates of protection, has hampered the development of effective dengue vaccines.

The lack of proper small animal models to study dengue pathogenesis unlike many other infections have significantly affected drug discovery and development of vaccines [58]. Mice and other small mammals are not infected with the naturally occurring DENV and therefore, dengue pathogenesis has predominantly studies by using mouse adapted DENV strains and immunodeficient mouse models, with the IFN type I and type II receptor deficient AG129 mice [59]. Humanized mouse models have also been developed [60], but are not commonly used due to the expense and also the variation is results and problems with reproducibility based on the human cell engraft [59]. The sylvatic strain of the DENV has shown to infect at least two different monkey species; the African green monkey and the Guinea baboon [61] and the Rhesus macaques are widely used as non-human primate models [62]. However, as none of the monkeys who are naturally infected, or the non-human primate models or the humanized mice show the features of vascular leak and severe dengue that occurs in humans, there are significant limitations in understanding dengue pathogenesis using animal models.

### Dengue vaccines for the prevention of dengue

There are several dengue vaccines which are in the process of being approved in different countries, and some are in phase 3 trials [63]. Only the first dengue vaccine, CYD-TDV, has been licensed by the WHO and has been used in mass immunization campaigns in Philippines and Brazil [64]. However, it was found to have poor efficacy against DENV serotype 2, and increased risk of hospitalization in young dengue naïve individuals and, therefore, was subsequently recommended to be given only to dengue seropositive individuals [64,65]. As CYD-TDV used a yellow fever virus backbone, it was thought that the lack of immune responses to DENV NS1 could have led to reduced efficacy in dengue naïve individuals and, therefore, other vaccines such as TAK-003, TV003/TV005 NIH vaccines contained the DENV virus backbone or the complete virus [63]. The efficacy of both CYD-TDV and TAK-003 in preventing virologically confirmed infection and hospitalizations is shown in Table 1.

**Table 1:** Comparison of efficacy rates for virologically confirmed dengue and for hospitalizations for CYD-TDV and TAK-003 3 years from the first dose as reported by Rivera et al [66] and Hadinegoro et al [67].

	CYD-TDV (aged >9 years) Efficacy % (95% CI)	CYD-TDV (aged <9 years) Efficacy % (95% CI)	TAK-003 Efficacy % (95% CI)
Overall efficacy virologically confirmed dengue	65.6 (60.7 to 69.9)	44.6 (31.6 to 55.0)	62 (56.6–66.7)
Baseline seropositive	81.9 (67.2 to 90.0)	70.1 (32.3 to 87.3)	65.0 (58.9–70.1)
Baseline seronegative	52.5 (5.9 to 76.1)	14.4 (–111 to 63.5)	54.3 (41.9–64.1)
Overall efficacy against hospitalization	80.8 (70.1 to 87.7)	56.1 (26.2 to 74.1)	83.6 (76.8–88.4)
Baseline seropositive	NA	NA	86.0 (78.4–91.0)
Baseline seronegative	NA	NA	77.1 (58.6–87.3)

NA indicates, not reported.

The TAK-003 vaccine is registered for use by the European Medicines Agency, Indonesia and Brazil [68]. The cumulative efficacy of this vaccine after three years in a phase 3 trial was observed to be 62% against virologically confirmed infection, 83.6% against hospitalizations and 65.4% against development of DHF, with the efficacy against hospitalization sustained at 70.8% at the end of the third year [66]. In a press release, the TAK-003 vaccine manufacturers stated that after 4.5 years, TAK-003 showed a 84.1% efficacy against hospitalization and 61.2% efficacy against virologically confirmed dengue, with no evidence of disease enhancement, which indicates that this vaccine is effective in significantly reducing hospitalizations and the overall burden due to dengue [69]. However, the TAK-003 vaccine did not demonstrate equal efficacies for all four serotypes, with 95.3% efficacy against hospitalizations in dengue seropositive individuals when infected with DENV2, and 69.2% and 72.1% when infected with DENV1 and DENV3, respectively at the end of three years [66]. The vaccine demonstrated no efficacy against DENV3 and DENV4 in DENV seronegative individuals against hospitalizations at the end of three years [66]. The vaccine demonstrated the highest efficacy (84.4%) against virologically confirmed dengue in Sri Lanka, which had DENV2 as the predominant circulating serotype during the trial period, whereas the efficacy rates in the Philippines, which had predominantly DENV-3, and Colombia, which predominantly had DENV-1, were 51.2% and 44.4%, respectively [66]. Therefore, it is important to study whether there is any waning of immunity especially to DENV-1 and DENV-3 and if these differences in overall efficacy for different serotypes matter. The WHO SAGE recently recommended the vaccine to be given in settings of high disease burden and high transmission rates to maximize public health impact and to minimize potential risks to seronegative individuals [70].

TV003 is a live attenuated tetravalent dengue vaccine currently undergoing phase 3 clinical trials [71]. Studies in human challenge models were carried out using a modified strain of DENV2 and the vaccine was found to provide complete protection against infection [72]. Neutralizing antibody responses for all four serotypes were seen in 48% of the subjects and phase I/II studies carried out in India showed that 77.8% to 81.9% of individuals seroconverted to the different DENV serotypes [73,74]. Although no efficacy data are currently available for this vaccine, based on observations from human challenge models, the vaccine appears to induce a high level of protection for at least a short duration; it will be interesting to see if these findings translate to similar results in large scale clinical trials over longer periods.

Although there are several promising dengue vaccine candidates, including those that are currently been registered in many countries (TAK-003), there appears to be some waning of immunity over time [75]. While all the current vaccine candidates are tetravalent live attenuated vaccines, it would be important to evaluate if mRNA vaccines could induce a DENV specific immune response of a higher magnitude for a longer duration. For instance, although many different SARS-CoV-2 variants are emerging and causing outbreaks in different regions [76], hospitalization rates and deaths have been very minimum in many countries, which only used one booster dose of a mRNA vaccine due to the lack of availability of mRNA vaccines for repeated boosters [7].

### Therapeutics for the treatment of dengue

In the absence of effective therapeutics, the management of patients infected with DENV is limited to symptomatic relief, judicious fluid replacement, and close monitoring for warning signs which may predict progression to severe dengue. Without specific biomarkers for plasma leakage, clinicians often rely on patient’s self-reported warning signs like abdominal pain or persistent vomiting, thorough physical examination including hemodynamic assessment, and careful interpretation of blood parameters such as rise in the hematocrit level and decline in platelet counts to guide a timely and adequate fluid replacement to prevent dengue shock syndrome [52]. It is crucial to tailor the fluid replacement according to the extent of leakage, to prevent prolonged hypotension and shock and also to prevent fluid overload [77]. As both prolonged shock and fluid overload is associated with high mortality rates, fluid replacement should be carefully monitored, to make sure the fluid replacement is initiated in a timely manner, for required duration with the correct type of fluid [77–79].

**Fig 2:**
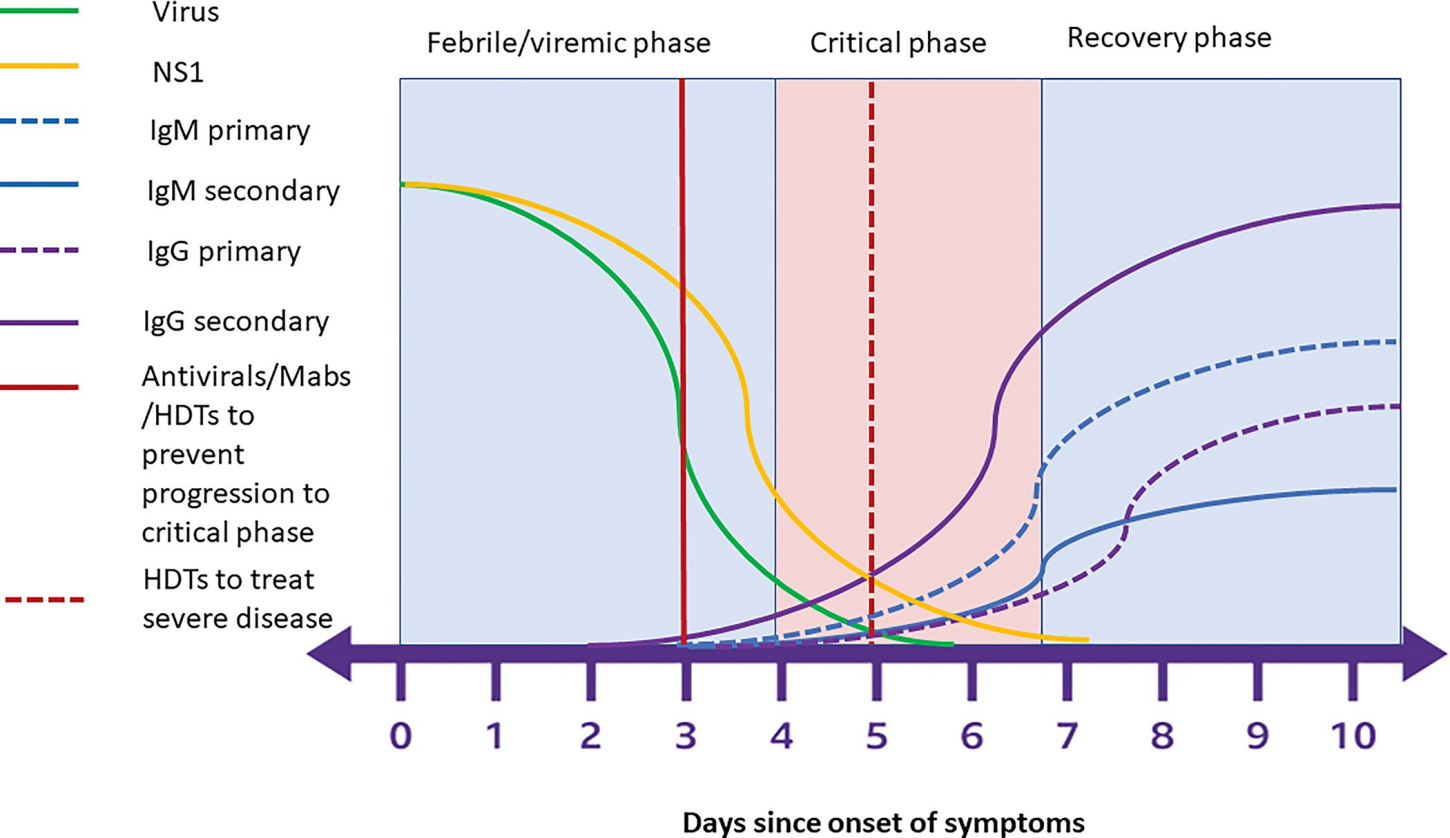
Changes in viral loads, NS1 antigen levels and the antibody responses to dengue during different clinical stages of dengue, and ideal timing of therapeutics. Antivirals, monoclonal antibodies that neutralize the virus (Mabs) or host-directed therapies (HDTs) that aim to prevent progression to severe disease, should be given before the patient proceeds to the critical phase of illness (around day 3 to 6), while HDTs that aim to treat severe dengue, could be given during the critical phase.

Therefore, even though dengue infections are mostly mild and self-limiting, many patients are routinely followed up in outpatient clinics daily from febrile phase until recovery phase. Patients who have uncontrolled comorbidity or exhibit warning signs are usually hospitalized preemptively for close monitoring and further management. While this standard of care will minimize the morbidity and mortality of dengue, the health care facilities in resource-poor countries could potentially be overwhelmed by the surge of patients during each seasonal dengue outbreak. Furthermore, all health care personnel are required to be regularly trained for early detection of complications, monitoring of patients and fluid management to reduce mortality rates. An ideal therapeutic option for dengue would, therefore, be a treatment that is safe, inexpensive, easy-to-administer, and widely available for patients seeking health care during early dengue illness to prevents progression to severe disease (Fig 2).

There have been many efforts to find safe and effective treatments for dengue, with studies to identify direct acting antiviral agents which reduce or inhibit viral replication and drugs that target the host. Many of the direct acting antivirals that have been evaluated targeted the envelope, capsid, NS5, NS2B/NS3 protease, NS4A and, in some cases, NS4B [80]. There have been many studies on host-directed antivirals, for example, those which inhibit viral entry by targeting CCR5, and those that target fatty acid synthetase, HMG-CoA reductase, ubiquitin-proteosome pathways and alpha-glucosidase [80–82]. Only a few of these compounds have progressed beyond preclinical studies, with celgosivir being evaluated in a phase 1b trial, and lovastatin and chloroquine being evaluated in phase 2 clinical trials [81–83]. More recently, new molecules targeting NS4B and NS5 have shown promising results *in vitro* and in dengue mouse models [84–86]. These novel compounds are currently undergoing phase 1b and phase 2 clinical trials, which are to conclude in 2023 and 2024. However, AT-725, an NS5 inhibitor developed by Atea that was undergoing phase 2 clinical trials, was terminated, due to the need for a larger sample size because of individual variability, the large costs involved in such trials and the lack of diagnostics with good sensitivity [87].

In addition to small molecules, use of DENV specific monoclonal antibodies have also been explored as potential therapies [88,89]. Most of these therapeutic antibodies have been directed at the envelope protein, and modified to prevent inducing ADE [88–90], while some therapeutic antibodies have been developed against NS1 [91,92]. However, only those which are targeting different epitopes or regions of the envelope protein have so far progressed beyond the preclinical phase and are currently being evaluated in phase 1 or 2 clinical trials [89,93]. While administering monoclonal antibodies as therapeutics for dengue in the large number of individuals who are infected with the virus would be costly, if such therapies, which can be given as a single dose, is given during early illness, it could be cost effective by reducing hospitalizations and the huge cost involved in outpatient monitoring of patients with dengue.

Although using antiviral drugs represent a rational approach to treating dengue infections and preventing progression to severe disease, severe dengue does not occur due to higher viral loads alone [94], and there is risk of viral escape. Furthermore, there are lower viral loads and earlier clearance of viraemia in secondary dengue, which is associated with a higher risk of severe dengue, than in primary dengue [95]. Vascular leak, a hallmark of severe dengue, occurs due to endothelial dysfunction, which coincides with a reduction in viraemia [96]. While viral proteins such as NS1 contribute to endothelial dysfunction, a dysfunctional innate immune response, mast cell degranulation, inflammatory cytokine and chemokine production by innate immune cells and increase in inflammatory lipid mediators can all play important roles [97–99]. Many different cytokines, chemokines, inflammatory lipid mediators and mast cell products are seen during early illness (in the first 72 hours) in dengue-infected individuals who progress to severe dengue [98,100–103]. Therefore, targeting the host response during early illness is potentially important for reducing progression to severe dengue and may carry less risk of viral escape.

Since NS1 engages with TLR4 resulting in release of proinflammatory cytokines and inflammatory lipid mediators, directly damaging the endothelial glycocalyx and contributing to vascular leak, antivirals are likely to prevent endothelial dysfunction induced by NS1 [104–106]. Drugs that inhibit mast cells or block mediators such as PAF, and leukotrienes that are released due to mast cell degranulation, have shown some efficacy in reducing complications due to dengue in preclinical studies and in clinical trials [107–110]. Therefore, as it is evident that the host immune response plays an important role in the pathogenesis of dengue, drugs that target these dysfunctional immune responses could be beneficial and it would be important to explore their efficacy in larger trials.

### Challenges in delivering a treatment

Dengue infections are associated with a relatively short viraemia with viral loads plummeting after 72 hours, especially in secondary dengue infections [95,111,112]. Therefore, antiviral drugs for dengue probably need to be given very early in the illness to prevent progression to severe disease. As adult patients present to a health care professional after 72 hours of fever in most instances, and the critical phase typically occurs later, a combination of an antiviral with host directed therapy may be preferable to using an antiviral treatment alone. However, in order to treat patients with dengue, reliable point-of care diagnostics which are cheap and have a high sensitivity to diagnose dengue at the time of presentation to a health care facility are also needed. Since millions of individuals experience symptomatic dengue each year, with millions being monitored for possible progression to severe disease, it is important that a treatment for dengue is extremely safe, affordable and accessible to those who need it most. The availability of a test to identify patients who are likely to progress to severe disease would be extremely beneficial to selectively treat such patients, or to prioritize such patients for treatment. A safe and affordable combination of an antiviral with a host directed therapy is likely to achieve the best therapeutic effect.

## Conclusions

The global burden of dengue is rising due to climate change, rapid and unplanned urbanization, improper waste management and co-circulation of multiple DENV serotypes. Due to changes in population demography in many dengue-endemic countries, this previously predominantly childhood infection is now causing a significant disease burden in adults, with a high risk of severe disease and higher mortality in pregnant women and in those with metabolic disease. Furthermore, as the clinical features of dengue mimics many febrile illnesses present in these regions and due to lack of point-of-care diagnostics of high sensitivity, identifying dengue patients during early illness is sometimes a challenge. Although all individuals living in dengue endemic countries are equally susceptible, marginalized populations are affected significantly more due to loss of livelihood and the indirect costs associated with dengue infections. In addition, due to the lack of a prognostic biomarker that can detect patients who progress to severe disease in early illness, all suspected patients are closely monitored to detect complications and initiation of fluid replacement. This further adds to the economic burden of many dengue endemic countries and leads to overwhelming of health care systems, especially during large dengue outbreaks.

There have been many innovations in controlling the vector, such as the release of *Wolbachia*-infected mosquitoes and spatial repellents, which have demonstrated success in some countries. In addition, a new dengue vaccine (TAK-003) has been registered by the European Medicines Agency, Brazil and Indonesia, and is likely to be registered in additional countries soon, which another vaccine, TV003 is undergoing phase 3 trials. However, as the vaccines are approximately 80% effective against prevention of hospitalization, there will still be a need for drugs to prevent progression to severe disease in many patients. While novel vector control methods and dengue vaccines are emerging, with the projected increase in the burden of dengue due to climate change and urbanization, a significant proportion of patients will still require hospitalization. Therefore, in addition to vector control methods and vaccines there should be a sustainable, integrated strategy for dengue control, which includes development of specific treatments, which has often been neglected. Efforts of drug development and identification of biomarkers has been hampered due to the incomplete data regarding pathogenesis of severe dengue and correlates of protection. There is an urgent need for a treatment for dengue to reduce progression to severe disease and thereby reduce hospitalizations and complications of dengue. It would be important that all such measures (vaccines, vector control and drugs), are equally accessible to marginalized populations who are affected most by dengue.
