# Correction: Facing the escalating burden of dengue: Challenges and perspectives

**DOI:** 10.1371/journal.pgph.0003499

**Published:** 2024-07-09

**Authors:** Gathsaurie Neelika Malavige, Peter Sjö, Kavita Singh, Jean-Michel Piedagnel, Charles Mowbray, Sergio Estani, Steven Chee Loon Lim, Andre M. Siqueira, Graham S Ogg, Laurent Fraisse, Isabela Ribeiro

The eighth author’s name is spelled incorrectly. The correct name is: Andre M. Siqueira.
